# COVID-19 Lockdown Measures: Impact On Older Adults With Dementia And Their Caregivers In Singapore

**DOI:** 10.1093/geroni/igab046.543

**Published:** 2021-12-17

**Authors:** Quin W Yow, Tharshini Lokanathan, Hui-Ching Chen

**Affiliations:** 1 Singapore University of Technology & Design, Singapore, Singapore; 2 Singapore University of Technology and Design, Singapore University of Technology and Design, Singapore

## Abstract

Family caregivers typically rely on community-based services and social support networks to supplement their caregiving of older adults with dementia (OAwD). In April 2020, the Singapore government implemented a partial lockdown to contain the spread of COVID-19. We assessed the policy's impact on the physical and mental health of caregivers and their OAwD. As part of a larger study, 30 caregivers were interviewed and rated the stress they experienced when caring for an OAwD during the partial lockdown. Qualitative analyses found significant behavioral changes in OAwD such as irritability, aggression and hallucinations, which led some caregivers to believe their dependents’ condition had deteriorated, as well as heightened levels of caregiving stress. Overall, our preliminary results suggested that although social distancing measures may be effective in containing the spread of COVID-19, these measures could lead to negative outcomes on vulnerable populations such as OAwD and their caregivers.

